# Functionalization of Carbon Nanomaterial Surface by Doxorubicin and Antibodies to Tumor Markers

**DOI:** 10.1186/s11671-016-1537-z

**Published:** 2016-06-29

**Authors:** Olena M. Perepelytsina, Olena M. Yakymchuk, Mychailo V. Sydorenko, Olga N. Bakalinska, Francesco Bloisi, Luciano Rosario Maria Vicari

**Affiliations:** Department of biotechnical problems of diagnostic IPCC, Nauky str.,42/1, Kiev, 03028 Ukraine; Chuiko Institute of Surface Chemistry NAS of Ukraine, 17 General Naumov str., Kiev, 03164 Ukraine; Dipartimento di Fisica, Università degli studi di Napoli Federico II, Piazzale Teccnio, Naples, 80-80125 Italy; SPIN-CNR, Via Cintia 1, Naples, 80124 Italy

**Keywords:** Ultra dispersed diamonds, Onion-like carbons, Doxorubicin, MCF-7, MAPLE deposition, Functionalization, 61. 46+w, 61.48+c, 61.48De, 87.15-v, 87.64-t

## Abstract

The actual task of oncology is effective treatment of cancer while causing a minimum harm to the patient. The appearance of polymer nanomaterials and technologies launched new applications and approaches of delivery and release of anticancer drugs. The goal of work was to test ultra dispersed diamonds (UDDs) and onion-like carbon (OLCs) as new vehicles for delivery of antitumor drug (doxorubicin (DOX)) and specific antibodies to tumor receptors. Stable compounds of UDDs and OLCs with DOX were obtained. As results of work, an effectiveness of functionalization was 2.94 % *w*/*w* for OLC-DOX and 2.98 % *w*/*w* for UDD-DOX. Also, there was demonstrated that UDD-DOX and OLC-DOX constructs had dose-dependent cytotoxic effect on tumor cells in the presence of trypsin. The survival of adenocarcinoma cells reduced from 52 to 28 % in case of incubation with the UDD-DOX in concentrations from 8.4–2.5 to 670–20 μg/ml and from 72 to 30 % after incubation with OLC-DOX. Simultaneously, antibodies to epidermal growth factor maintained 75 % of the functional activity and specificity after matrix-assisted pulsed laser evaporation deposition. Thus, the conclusion has been made about the prospects of selected new methods and approaches for creating an antitumor agent with capabilities targeted delivery of drugs.

## Background

Nanotechnology enables to carry out controlled manipulation with substances at the level of 1 nm. That actually means to control physical, chemical, and biological processes at atomic and molecular scale [1–6]. Methods and vehicles are created for targeted transportation of drugs and biologically active substances in the body and active diagnostic devices for express analysis. It can be used for biotechnology, pharmaceuticals, and medicine purpose [7–13]. One of the focuses of the anticancer investigations is a search for effective antitumor drugs by inhibition of tumor cell proliferation, metabolism, and metastatic activity [14, 15]. Doxorubicin is one of such inhibitors [16–19]. Doxorubicin is an anthracycline drug which was extracted from *Streptomyces peucetius* var. caesius in the 1970s. There are two proposed mechanisms by which doxorubicin acts in the cancer cell: (i) intercalation into DNA and disruption of topoisomerase-II-mediated DNA repair and (ii) generation of free radicals and their damage to cellular membranes, DNA, and proteins. In brief, doxorubicin is oxidized to semi-quinone, an unstable metabolite, which is converted back to doxorubicin in a process that releases reactive oxygen species. Reactive oxygen species can lead to lipid peroxidation and membrane damage, DNA damage, and oxidative stress and triggers apoptotic pathways of cell death [20]. According to the classification of chemotherapeutic agents by mechanisms of action, doxorubicin is referred to antimetabolites as far as it can intercalate with DNA and cytotoxic antibiotics of anthracycline family because it affects topoisomerase II enzyme [21]. As a result, doxorubicin significantly reduced the proliferation and survival of tumor cells. However, the cytotoxic activity of doxorubicin has no specificity, which leads to serious side effects of the gastrointestinal tract, liver, and kidneys. It is noteworthy that such side effects inherent to the action of many anticancer drugs. We maintain opinion that solution is in usage of specific polymer materials which combine function of drugs vehicle and holder of antibodies to specific receptors of tumor cells. The possibility for receptor-dependent influence on tumor cells and targeted delivery of antitumor agent to cells with specific receptor profile is very attractive and promising area of anticancer research [22–25]. Based on earlier studies, there was proposed the hypothesis of creating carbon-protein constructs for targeted delivery of drugs, growth factors, and biologically active substances on the base of carbon nanomaterials (CNMs). As a biologically inert basis for accession drug and tumor-specific antibodies, we propose ultra dispersed diamonds (UDDs) and onion-like carbons (OLCs) [1, 3, 10]. Thus, the goal of our work was to syntheses antitumor nanocarbon-protein conjugates (NCPCs) on the basis of carbon “nucleus” (UDDs or OLCs) with specific antibodies to the tumor-specific receptor of epidermal growth factor (EGFR) and antimetabolic anthracycline drug (doxorubicin (DOX)). The novelty of investigation idea is in combination of anti-proliferation properties of DOX and receptor—specific binding of antibodies to EGFR for targeted increasing concentration of DOX in tissue niches which over-expressed of EGFR. In such way, effectiveness of the antitumor treatment will be increased and level of hum full side effect will be minimized. As a biologically inert vehicle for accession DOX and anti-EGFR antibodies, we propose to use UDD or OLC aggregates. Then, methods of controlled releasing of DOX were tested. Due to estimated cellular responses on different concentrations of CNMs, DOX, NCPCs, MCF-7, and HT29 cells, viability was measured. Afterwards, activity of antibodies to EGFR after matrix-associated pulse laser evaporated (MAPLE) deposition on carbon surface was analyzed. In the results obtained, NCPCs allowed to realize sustained release of DOX and demonstrated excellent dose-dependent cytotoxicity to tumor cells and biocompatibility in inactivated form. So, these NCPCs with DOX represent a platform for targeted delivery and for cell-specific release of antitumor drugs.

## Methods

### Cell Lines

Breast adenocarcinoma cell line MCF-7 and hepatocellular carcinoma HT29 was kindly presented by the bank of cell lines of man and animals R.E.Kavetskiy’ Institute of Experimental Pathology, Oncology and Radiobiology of NAS of Ukraine. Cells were incubated under standard conditions in 5 % of CO_2_ and 100 % humidity in RPMI-1640 medium (Sigma, USA). Full medium was supplemented with 10 % fetal bovine serum (FBS, Sigma, USA) and 40 mg/ml gentamycin (Sigma) for cell culture. Serum-free medium was not supplemented with FBS. Cells were cultured in sterile 96-well plates, 6-well plates, 25-cm^2^ flasks (Nunc, Denmark).

### Purification of the CNMs

Purification of UDDs and OLCs from the metal/catalyst was performed by HF treatment. Elimination of amorphous carbon from carbon nanomaterials was realized by oxidation in air at 450–500 °C. This purification method is very simple: the crude sample containing UDD and OLC reacted with oxygen (from air) and created carbon dioxide or carbon monoxide. Some amount of carbon deposit has been obtained after HF (aqua) dissolution of the catalyst support were oxidized in air at 450–500 °C. An air flow quartz tubular reactor was used for this procedure for 130–150 min. We analyzed and described physical and chemical characteristics of obtained UDD and OLC in previous work [26].

### Oxidation of UDDs and OLCs

On the next step, the purified carbon nanomaterials (CNMs) were oxidized in 70 % HNO_3_ at 99 °C for 4 h and then washed with distilled water and 10 % NH_4_OH solution for 12 h. As a result, carbon nanomaterial oxides: ultra dispersed diamond oxides (UDDox) and onion-like carbon oxides (OLCox), were synthesized. After that CNMs were washed thrice with dH_2_O to neutral pH. The acidic sites on CNM surface were regenerated with 0.1 M HCl solution. The resulting oxidized CNMs-ox were separated by centrifugation, rinsed extensively, and re-suspended in DI water.

### Preparation of CNM-DOX Particles

**Fig. 1:**
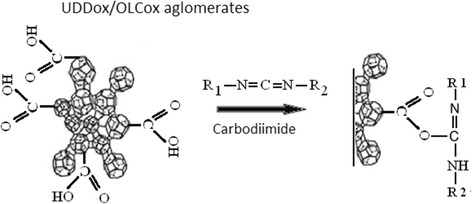
A scheme shows the mechanism of carboxyl group activation with carbodiimide. The reaction proceeded at room temperature during 15 min

**Fig. 2:**
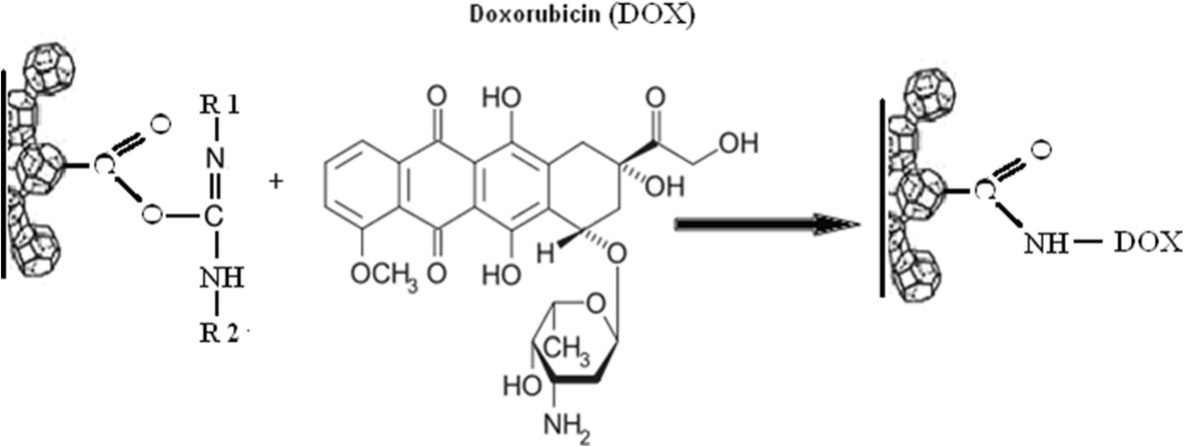
A scheme shows the mechanism of binding doxorubicin to UDD or OLC surface by protein covalent links. Reagents interacted at 30 °C for 24 h

The task of the next step was to immobilize DOX hydrochloride (DOX, Teva Nederland B.V, Netherlands) on the surface of UDDox or OLCox particles (UDD-DOX or OLC-DOX). To functionalize the surfaces of oxidized CNMs and UDDox/OLCox (200 mg) by amino containing DOX-lactose monohydrate, they were incubated with bifunctional linking agent—*N*-cyclohexyl-*N*′-(2-morpholinoethyl) carbodiimide metho-p-toluenesulfonate (C 1011, Sigma Aldrich, USA) during 15 min at room temperature (Fig. 1). After that, 58 mg DOX-lactose monohydrate was added into 10 ml of 0.15 M phosphate buffer pH 6.5 to each nanocarbon materials. The mixture was allowed to react at 30 °C for 24 h. In such conditions, covalent binds between DOX and CNMs were formed (Fig. 2). The composition were collected by centrifugation at 10,000 rpm 15 min and washed with dH_2_O thrice. Supernatants were used for detection concentration of free DOX by spectrophotometer.

### FT-IR Spectroscopy of UDD-DOX Particles

The chemical composition of the obtained composition was investigated by FT-IR spectroscopy. IR spectra were determined by FTS 7000e Varian FTIR spectrometer. Samples for analysis were prepared by grinding in a mill of a mixture of ~1 mg of nanomaterials and 150 mg of spectrally pure KBr. Samples were prepared by using a press with a pressure force of 3.0–3.5 × 10^3^ kg/cm^2^. Pre-shot spectra of KBr were preliminary obtained, and then, they are subtracted from the spectra of the samples. All spectra of UDD, DOX, and UDD-DOX were recorded by the same technology. All conditions of handling, sample preparation, and compositions were similar for the all substances.

### Colorimetric Assay

To monitor the amount of free DOX after immobilization on CNMs and the effectiveness of the DOX release, the ability of free DOX to fluorescent at a wavelength of 495 nm was used [27]. Active concentration of DOX was 16.7 % *w*/*w* from 3.34 mg/ml to 16.3 μg/ml. For the purpose of drawing the calibration lines, DOX Teva 20 mg/ml with ten dilutions to 8 × 10^−3^ mg/ml was used. Then, the fluorescence of free DOX was measured in supernatant from complexes DOX with UDD and OLC by spectrophotometric plate reader Multyscan (Labsystem, Finland).

### DOX Concentration in UDD-DOX and OLC-DOX Particles

For DOX immobilizations, 25 mg of UDDox or OLCox was used. The amount of DOX-TEVA was 2.25 mg of (0.75 mg of active DOX) in 1 ml dH_2_O. The amount of free DOX in supernatant after reaction was 14.3 μg or 1.9 % for OLC-DOX and 10.2 μg or 1.4 % for UDD-DOX. Thus, we made the conclusion that 735.7 μg of DOX was immobilized on 25 mg of OLCox and 739.8 μg of DOX was immobilized on 25 mg of UDDox in 1 ml dH_2_O. Effectiveness of functionalization was 2.94 % *w*/*w* for OLC-DOX and 2.98 % *w*/*w* for UDD-DOX. Further calculation of concentrations UDD-DOX and OLC-DOX are based on these data.

### MAPLE Deposition of Antibodies to EGFR

**Fig. 3:**
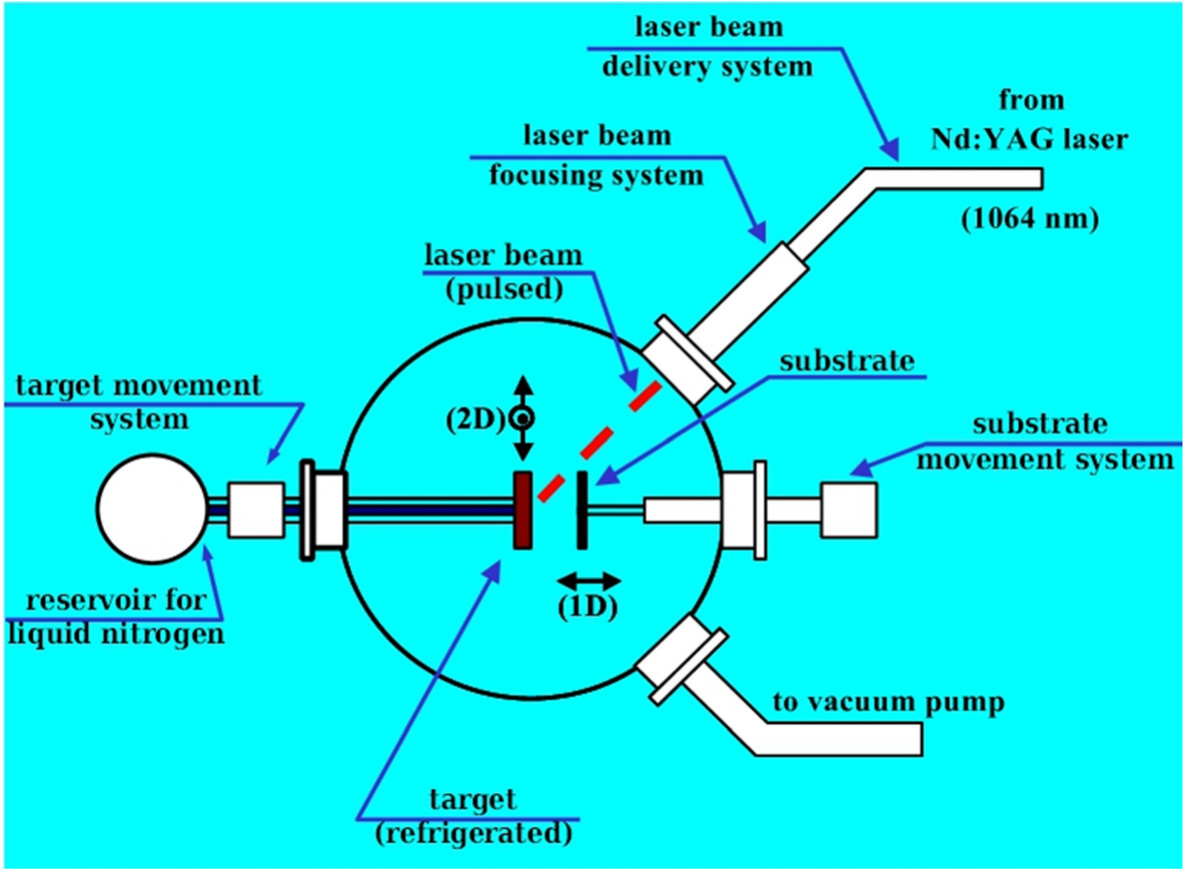
A scheme of matrix-assisted pulsed laser evaporation techniques shows principal of protein deposition on carbon surface. Antibodies to human epidermal growth factor receptor and epidermal growth factor were placed on target platen and evaporated on substrate. As substrates UDD-DOX (samples C46, C47) or OLC-DOX (samples C36, C37), films were used

MAPLE (Fig. 3) is a deposition technique [28] derived from pulsed laser deposition (PLD) in order to avoid laser radiation-induced damage to biologic or organic molecules or compounds. Such a result is achieved carrying out laser deposition from a frozen target containing the guest material to be deposited in a volatile matrix [29–32]. As specific MAPLE characteristic, the presence of the matrix protects guest material from being damaged by laser radiation but deposition is essentially solvent-free since matrix molecules do not reach substrate. This allows multilayer depositions using the same solvent and depositions on soluble substrates. The steps for production of antibody-CNM-DOX nanoparticles were as follows:

- A soluble layer was created on a MAPLE substrate (a microscope cover glass) by placing it at the bottom of a 5-mm deep container filled with sterile saline solution (9 mg/ml NaCl in water from Fresenius Kabi) and letting water to evaporate at 95 °C and atmospheric pressure for about 12 h.
- A layer containing the required nanoparticles (OLC-DOX or UDD-DOX, see Table 1 for details) was deposited on the top of the soluble layer by taking advantage of the solvent-free behavior of MAPLE technique. The MAPLE target was obtained by dispersing nanoparticles in aqueous or saline solution. The target was frozen by placing it in thermal contact with liquid nitrogen. The vacuum chamber pressure was then lowered and laser turned on. Laser beam impinges at an angle of 45° on the target which, during the deposition, is moved by a computer controlled translation system in order to avoid overheating and drilling: an area of about 2 cm^2^ is scanned, one or more times depending on the total number of pulses, during deposition time.

**Table 1:** MAPLE deposition parameters

Sample	C36	C37	C46	C47
1st MAPLE deposition				
Guest	OLC-DOX	OLC-DOX	UDD-DOX	UDD-DOX
Matrix	Saline solution 7.2 mg NaCl/1.0 ml H_2_O	Saline solution 7.2 mg NaCl/1.0 ml H_2_O	Water	Water
Target	10 mg guest/1.0 ml matrix	10 mg guest/1.0 ml matrix	10 mg guest/1.0 ml matrix	10 mg guest/1.0 ml matrix
Pulse energy	102 mJ/pulse	102 mJ/pulse	191 mJ/pulse	123 mJ/pulse
Number of pulses	4400 pulses	4400 pulses	8800 pulses	8800 pulses
Target to substrate	18 mm	10 mm	18 mm	10 mm
2nd MAPLE deposition				
Guest	Monoclonal rabbit anti-EGFR (clone SP9, RMPD 020, Diagnostic BioSystems, USA)	Monoclonal mouse anti-EGF (clone 2 F1, WH0001950M1, Sigma, USA)	Monoclonal rabbit anti-EGFR (clone SP9, RMPD 020, Diagnostic BioSystems, USA)	Monoclonal mouse anti-EGF (clone 2 F1, WH0001950M1, Sigma, USA)
Matrix	Saline solution 9.0 mg NaCl/1.0 ml H_2_O	Saline solution 9.0 mg NaCl/1.0 ml H_2_O	Saline solution 9.0 mg NaCl/1.0 ml H_2_O	Saline solution 9.0 mg NaCl/1.0 ml H_2_O
Target	2.0 ml guest/8.0 ml matrix	2.0 ml guest/8.0 ml matrix	2.0 ml guest/8.0 ml matrix	2.0 ml guest/8.0 ml matrix
Pulse energy	263 mJ/pulse	191 mJ/pulse	360 mJ/pulse	191 mJ/pulse
Number of pulses	8800 pulses	4400 pulses	8800 pulses	4400 pulses
Target to substrate	18 mm	10 mm	18 mm	10 mm
Both MAPLE depositions				
Target scanned area	1.7 cm^2^	1.7 cm^2^	1.7 cm^2^	1.7 cm^2^
Laser type	Q-switched	Q-switched	Q-switched	Q-switched
	Nd:YAG	Nd:YAG	Nd:YAG	Nd:YAG
Laser wavelength	1064 nm	1064 nm	1064 nm	1064 nm
Pulse duration	10 ns	10 ns	10 ns	10 ns
Pulse repetition rate	4 pulses/s	4 pulses/s	4 pulses/s	4 pulses/s
Spot size (elliptical)	1.0 mm × 1.4 mm	1.0 mm × 1.4 mm	1.0 mm × 1.4 mm	1.0 mm × 1.4 mm
Target temperature	145 K	145 K	145 K	145 K
Chamber pressure	10^−4^ Pa	10^−4^ Pa	10^−4^ Pa	10^−4^ Pa
- The soluble layer covered by a second layer containing OLC/UDD-DOX nanoparticles was used as a substrate for a second MAPLE deposition of anti-EGFR antibodies. The target was a saline solution of EGFR or EGR, the guest compound, frozen by thermal contact with liquid nitrogen. The MAPLE deposition (deposition parameters are detailed in Table 1) ensures again that previous layers were not damaged or removed.
- Finally, the microscope cover glass with the resulting multilayer deposition over soluble substrate was immersed in a saline solution as solvent. The soluble substrate dissolved and the nanoparticle layer disintegrated into individual antibody-OLC/UDD-DOX units.

### Cytotoxicity of CNMs

The cytotoxicity of DOX, UDD-DOX, and OLC-DOX was evaluated against MCF-7 and HT29 tumor cell using MTT assay. MTT test based on conversion of 3-[4,5-dimetltiazol-2]-2,5-dipheniltetratetrazolium salts to farmasan crystals by NAD(P)H-dependent mitochondrial oxidoreductase enzymes in alive cells. Protocol was described by T. Mosmann [33]. In brief, 1 × 10^4^ MCF-7 or HT29 were seeded in 96-well plates and cultured in full culture medium for 12 h. Then, current culture medium was replaced by culture medium containing DOX, UDD-DOX, and OLC-DOX. Cells which were cultured in full medium were used as control. After 24 h of incubation, cells were analyzed with (MTT) by colorimetric assay. To 100 μl of cells suspension, we added 20 μl MTT solution (5 mg/ml PBS, Sigma). After that, cells were incubated with MTT during 4 h in standard conditions. Then, samples were centrifuged under 1500 g, during 5 min, and supernatant was extracted. In all, wells were added 10 μl DMSO (Sigma) for MTT crystals dilution and 20 μl of 25 mM glycine. The absorbance of reacted solution was measured at 540 nm on spectrophotometric plate reader Multyscan (Labsystem, Finland). The cell viability in the tested concentration range of CNMs was determined by the following equation:

$$ \mathrm{Cell}\ \mathrm{viability},\ \% = \left(\mathrm{Absorbance}\ \mathrm{of}\ \mathrm{test}\ \mathrm{group}\right) \times 100/\left(\mathrm{Absorbance}\ \mathrm{of}\ \mathrm{control}\ \mathrm{group}\right). $$

Three replicates were used for control and test groups. Absorbance for each sample was determined three times.

### Sandwich ELISA

**Fig. 4:**
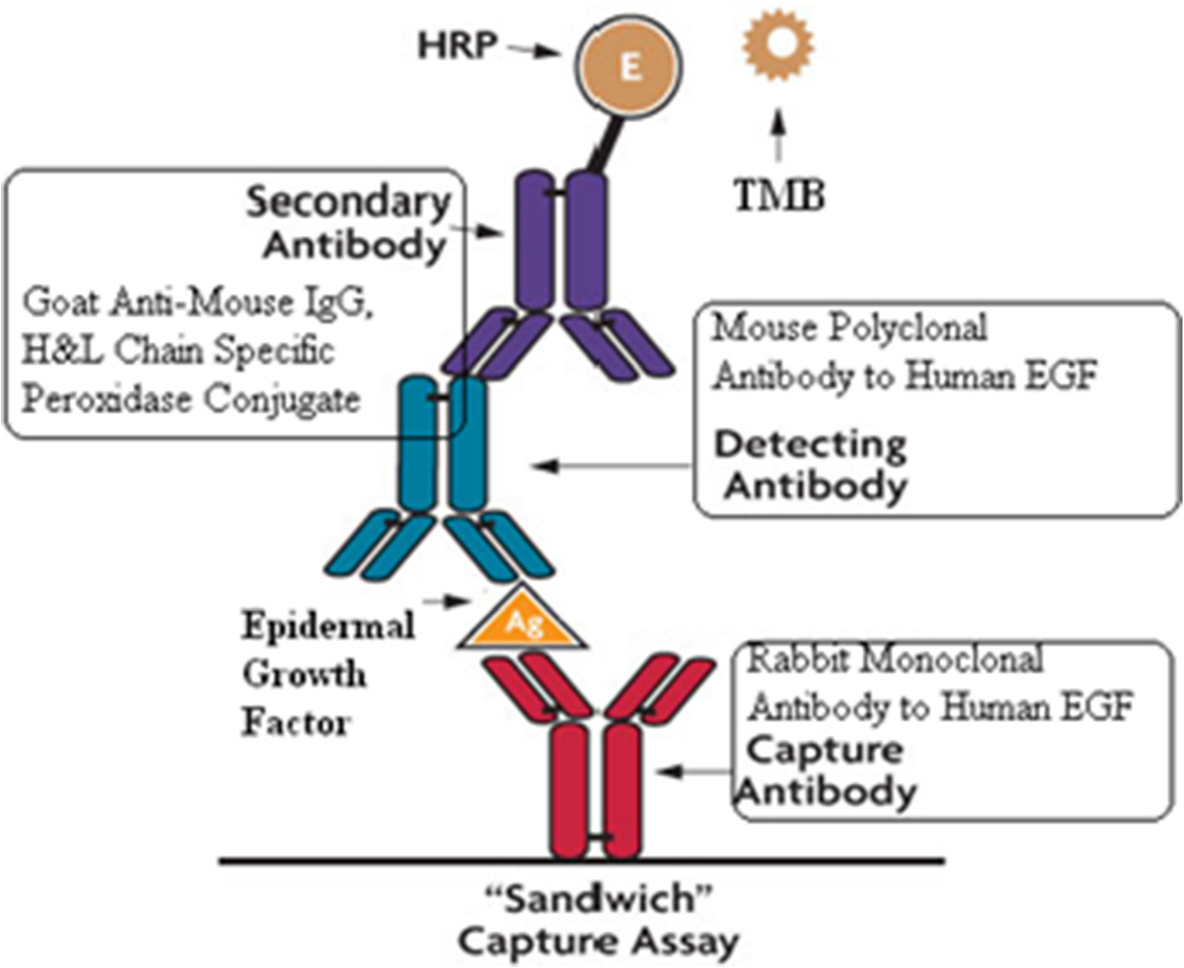
Scheme of sandwich ELISA method

The sandwich enzyme-linked immunosorbent assay (ELISA) measures the amount of antigen between two layers of antibodies (i.e., capture and detection antibody). The antigen to be measured must contain at least two antigenic sites capable of binding to antibody, since at least two antibodies act in the “sandwich” (Fig. 4). In this work, protocols which were recommended by producers of antibodies Sigma®, USA and Calbiochem®, Germany, were used. As Capture antibody, we used rabbit monoclonal anti-epidermal growth factor (EGF) antibody (SAB 2104809, Sigma, USA). As detection antibody, mouse polyclonal anti-EGF (clone 2 F1, WH0001950M1, Sigma, USA) was used. For visualization, as secondary antibody, goat anti-mouse IgG peroxidase conjugate (№ 401253, Calbiochem, Merck, Germany) was chosen. EGF human recombinant, purchased by Sigma, USA (SRP 3027, Sigma, USA), was used as an antigen. As substrate, we used 3,3,5,5-tetramethylbenzidine (TMB, № 613544, Calbiochem, Merck, Germany). Detecting antibody in ×10 concentration was object of MAPLE deposition on UDD-DOX or OLC-DOX films (C37 and C47 samples). For MAPLE deposition in samples C36 and C46, rabbit monoclonal anti-EGF-r antibody (clone SP9, RMPD020, Diagnostic Biosystems, USA) was used. After MAPLE deposition of the antibodies on the substrate form saline and water solution of UDD/OLC-DOX particles, the samples were immersed in saline (1 ml) and resolved by incubation on a rotary shaker (80 rpm) for 24 h at room temperature. Then, the activity of antibody were tested by ELISA procedure as a reaction immunoassay as detecting antibody.

### Statistical Analysis

Section of statistics, dedicated to processing of small samples (2 ≤ n <20), conventionally called micro-statistical. Underlying micro-statistical estimates of normally distributed random variables is the Student’s distribution for small quantities. Reported *p* values was *p < 0.05 or **p < 0.01.

## Results and Discussion

### FT-IR Spectroscopy of UDD-DOX Particles

**Fig. 5:**
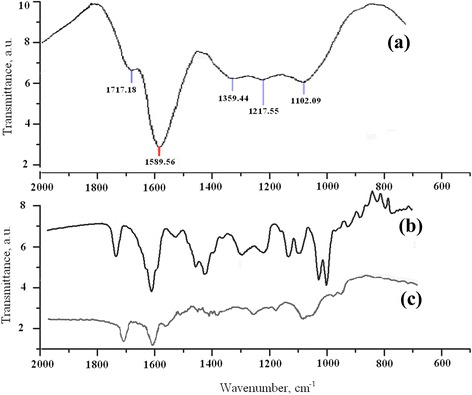
FT-IR spectra of UDD (**a**), DOX (**b**), and UDD-DOX (**c**)

Figure 5 shows FT-IR spectra of UDD, DOX, and UDD-DOX particles. The main features of UDD (Fig. 5a) correspond to existing quite intense peaks of carbohydrate linkages in the region of 1717 cm^−1^. It indicates the presence of carboxyl groups. There was also a shift and decrease in intensity of the C–O covalent band vibrations, which were fixed at 1619–1589 cm^−1^. Also, in samples, low vibration peaks of aromatic C–H groups (792 cm^−1^) were demonstrated. Meanwhile, some carbonyl groups can emerge from adsorbed CO and CO_2_ (at 1102 and 1247 cm^−1^, respectively) in UDD spectra. The appearance of a vibration at 1359 cm^−1^ is evidence of the presence in UDD of cyclic carbon groups. Figure 5 demonstrates FT-IR spectra of DOX (b) and UDD-DOX particles (c). It shows characteristic picks from UDD and DOX even after several cycles of washing and centrifugation of nanomaterials.

### In Vitro DOX Release from CNMs-DOX Particles by Trypsinization

**Fig. 6:**
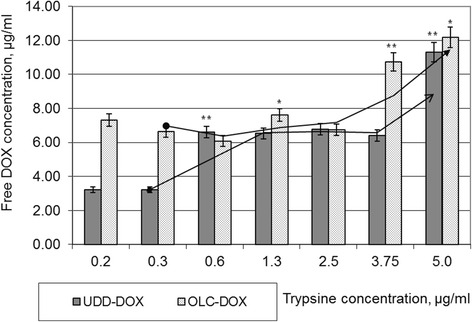
Cleavage of UDD-DOX and OLC-DOX conjugates and release of free doxorubicin upon incubation with trypsin at 37.0 °C in dark during 24 h. Each experiment was carried out in triplicates. Concentration of free doxorubicin was calculated by spectrophotometer absorbance and calibration lines at 495 nm

The treatment of the oxidized carbon nanomaterial (UDDox, OLCox) solutions in water soluble carbodiimide hydrochloride and DOX led to formation of covalent peptide bonds between DOX and carbon surface. Such bonds are strong at phosphate-buffered saline (PBS, pH 7.7) at temperatures ranged of 35.0–37.0 °C. Therefore, controlled release of DOX was the task of the next step of our work. For decomposition of UDD-DOX and OLC-DOX conjugates and DOX release, we used trypsin. Trypsin is one of the best characterized serine proteases [34]. The concentration of trypsin in our study was 0.25 mg/ml (0.25 *w*/*v*) in serum-free DMEM. The trypsin as pancreatic enzyme is involved in hydrolysis of peptide bonds by sites of carboxyl groups of serine amino acids. The UDD-DOX and OLC-DOX particles in a concentration of 650 μg/ml were incubated with trypsin at concentrations from 0.2 to 5.0 μg/ml at 37.0 °C in dark during 24 h. The concentration of attached DOX was 20 μg/ml. After incubation, the 96-well plates were centrifuged. Supernatant from wells was filtered through a filter with a pore size of 0.22 μm. The absorbance of supernatant was measured using a spectrophotometric plate reader at the wavelength 490 nm. The amount of free DOX was calculated according to the calibration line. As a result, it was found dose-dependent increasing of the concentration of free DOX after incubation with trypsin (Fig. 6). Interestingly, that correlation between concentration of trypsin and free DOX is positive but is not proportional. DOX release has stepped-like tendency. For example, for the UDD-DOX complex, at trypsin concentrations of 0.2–0.3 μg/ml, the concentration of the released DOX was about 3.22 μg/ml; at trypsin concentrations 0.6–3.75 μg/ml, concentration of the released was equal 6.5–6.8 μg/ml; and at trypsin concentration 5.0 μg/ml, free DOX concentration has risen to 11.31 μg/ml. For complex OLC-DOX, also, observed stepwise trends of growth the concentration of free DOX. At trypsin concentrations from 0.2 to 0.6 μg/ml and 1.3 to 2.5 μg/ml, changing in concentration of DOX was statistically insignificant (6.0–6.6 and 7.6–7.7 μg/ml, respectively). In the case of trypsin concentration 5.0 μg/ml, the concentration of the free DOX has reached 12.18 μg/ml. Thus, it can be assumed that trypsin can destroy the covalent peptide bonds between DOX and the carbon surface and facilitate controlled release of the active substance. More than that, the efficiency of such peptidase action as destruction of covalent peptide bonds and the amount of released DOX depends on specificity of the three-dimensional structure of the carbon substrate.

### Placement of Nanomaterials, DOX, and Antibodies on Cover Slips

**Fig. 7:**
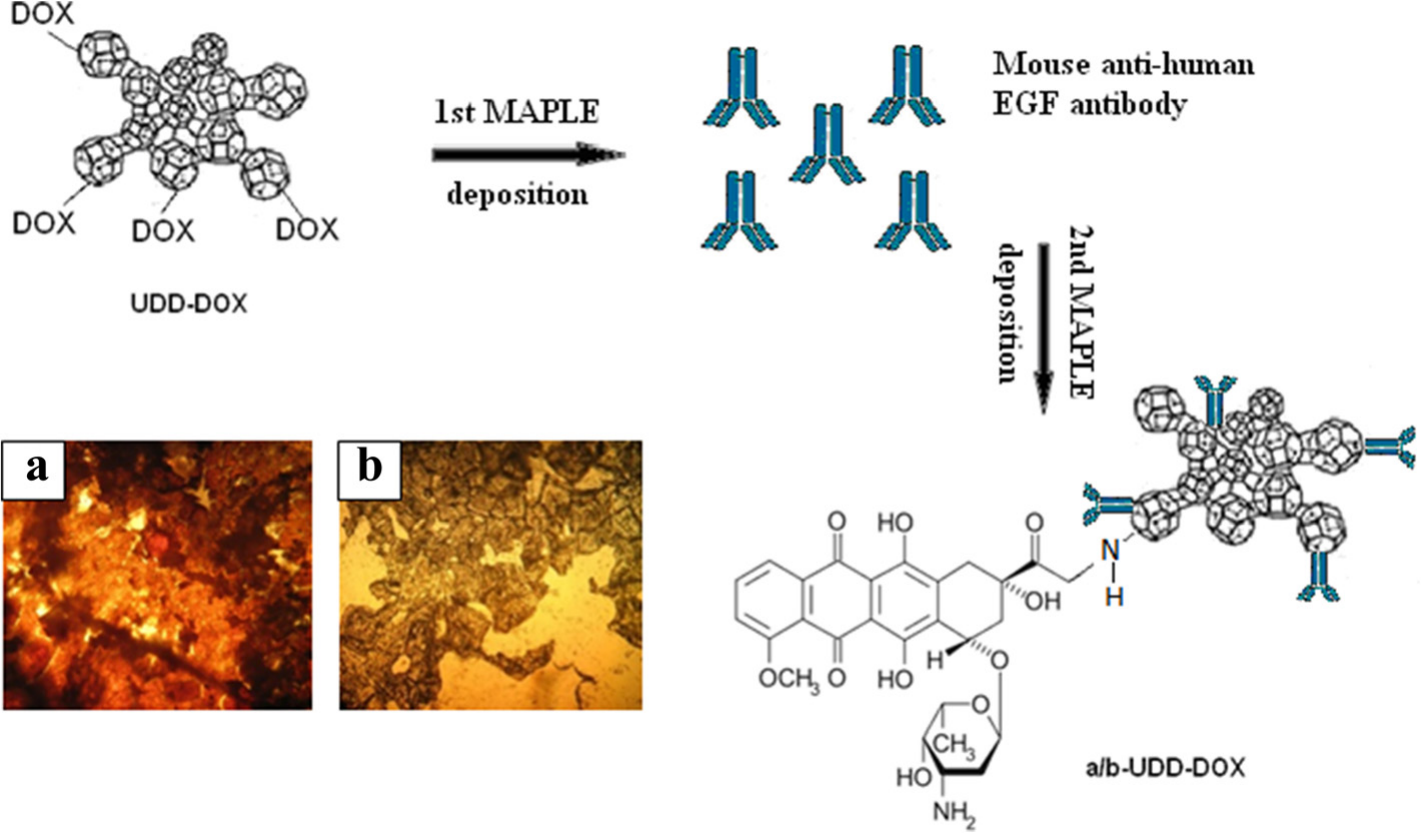
A schematic drawing shows structure of EGF-antibody-UDD-DOX conjugates. Microphotography of carbon-protein films after MAPLE deposition. Images were acquired using Zeiss Flouval ×40 water immersion lens. **a** C36 sample, saline solution of UDD-DOX-rabbit antihuman EGFRa/b. **b** C37 sample, saline solution OLC-DOX-mouse antihuman EGFa/b

Another problem which considerably limits the usage of anticancer drugs is non-specific toxicity. Chemotherapy is associated with significant drug leakage into systemic circulation and non-specific distribution to healthy tissue. In results, chemotherapy correlates with severe cardiac, neuronal, hepatic, kidney, and bone narrow toxicity [35, 36]. Therefore, the task of targeted delivery of anticancer drugs still remains actual, and it is not solved yet. Application of tissue-specific antibody is seemed as one of the possible ways to resolve this task. Tumor cells of breast cancer (MCF-7 cell line) are characterized of high secretion of EGF and over expression of EGF receptors. EGFR is widely used as a marker in diagnostic of malignant growth. That is why, EGFR is a promising target for specific delivery of chemotherapeutic agents. With this mind, the task was to find a method of placement of complex protein molecules on carbon surface and at the same time not to destroy it tertiary structure and functional activity. Therefore, MAPLE technology was used for deposition of antibodies to EGFR on UDD-DOX or OLC-DOX surface (Fig. 7).

### Verification of Antibody to EGF Activity by ELISA

In order to determine the activity of antibodies after MAPLE deposition procedure on the surface of the carbon, we used two types of antibodies: C36 and C46—antibody to human EGFR from rabbit and C37 and C47—antibody to human EGF from mouse (Table 2). Activity of all samples analyzed by sandwich ELISA.

**Table 2:** Description of antibody in samples for MAPLE deposition

Sample	C36	C37	C46	C47
2nd MAPLE deposition	Application: immunohistochemistry	Application: ELISA	Application: immunohistochemistry	Application: ELISA
Guest	Monoclonal rabbit anti-EGFR	Monoclonal mouse anti-EGF	Monoclonal rabbit anti-EGFR	Monoclonal mouse anti-EGF

**Fig. 8:**
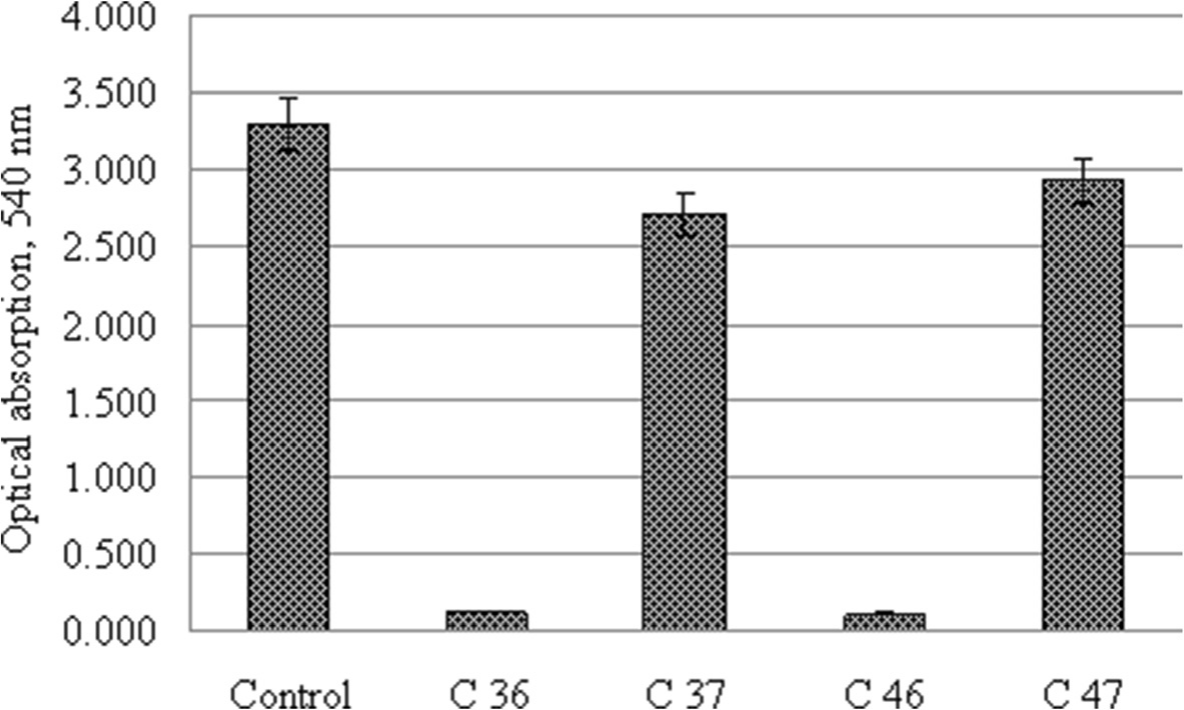
Activity of anti-EGF antibodies after MAPLE deposition in sandwich enzyme-linked immunosorbent assay

Samples C36 and C46 were used as a negative control, since they did not participate in ELISA reaction. Mouse antibodies to EGF which did not pass through MAPLE placement process were used as control sample. Human EGF was used as active substrate in ELISA assay. In results, binding activity of anti-EGF antibodies decreased slightly after placement in samples C37 and C47. Activity of antibody decreased by 18–22 % in C36 samples compared with the control. In C46 samples, activity of antibody decreased by 11–12 % (Fig. 8). At the same time, negative control non-specific binding in C36 and C46 samples provided 3 % of experimental error. It is possible that non-specific binding appears due to bovine serum albumin in ELISA protocol and illustrates the degree of reliability of the experimental deviations from the control samples. Nevertheless, the resulting antibody activity in C37 and C47 samples is quite high, about 75 % after MAPLE deposition. Samples C36 and C46 will be used for follow-up test of activity of antibodies after MAPLE placement in immunohistochemistry staining in further experiments. Authors are aware that only after a comprehensive analysis by different methods, we can make a conclusion about effectiveness of proposed method for placing the antibodies on the surface of the carbon films.

### Cytotoxicity of Carbon-DOX Conjugates after Incubation It with Trypsin

**Fig. 9:**
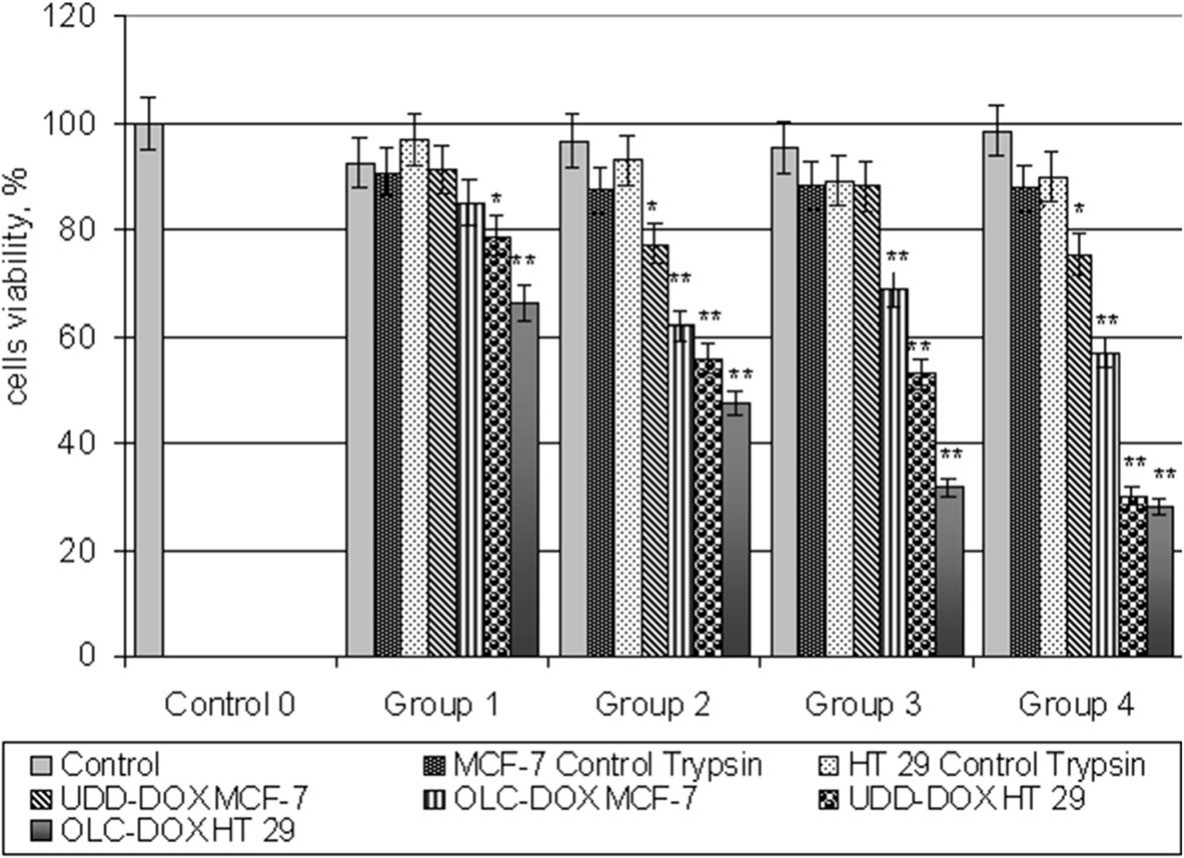
The survival of tumor MCF-7 and HT29 cells in concentration of 1 × 10^4^ cell/ml after incubation with the carbon-protein complexes UDD-DOX and OLC-DOX in the presence of trypsin. Cells were incubated in full medium 24 h and in presence of trypsin in serum-free medium next 24 h. Cell survival was evaluated by MTT assay

Cytotoxicity of DOX after releasing from the NCPCs was evaluated in vitro on monolayer cell cultures of hepatocellular carcinoma (HT29) and breast adenocarcinoma (MCF-7). For test, nine groups were formed (Table 3). 1 × 10^4^/well tumor cells (MCF-7 or HT29) were seeded in a 96-well plate. The cells were incubated in full medium during 24 h. After that, medium was changed for serum-free and trypsin. Then, UDD-DOX or OLC-DOX was added to cell culture. After 24 h of cultivation at standard condition cells, viability was measured by MTT assay. For estimation of possible cytotoxic influence of pure trypsin in experiments, trypsin control groups were added. Concentrations of trypsin in serum-free DMEM were demonstrated in Table 3. Figure 9 shows that even high concentrations of trypsin (125,250 μg/ml) did not lead to statistically significant effect on tumor cell viability. It is possible, because period of cultivation was rather brief. Another explanation to this fact is in increased resistance of tumor cells both to lack of nutrients (FBS) and to cytotoxic agents (trypsin). At the same time, it was found that increasing the concentration of trypsin and NCPCs correlated with decreasing of tumor cell survival. In group 1 at minimum concentration of trypsin (31.25 μg/ml) and NCPCs (84–2.5 μg/ml), level of tumor cell viability was the highest (from 90 to 66 %, depends on group). On the other hand, the increasing concentration of NCPCs and trypsin led to decreasing of tumor cell viability in both cell lines. It is worth to note that sensitivity of tumor cells depended on concentration of active ingredients and type of carbon in conjugates. Overall UDD-DOX conjugates were more cytotoxic than OLC-DOX. In addition, breast adenocarcinoma cells, MCF-7, were more sensitive to the action of the NCPCs-DOX (as to UDD-DOX as to OLC-DOX) than HT29 cells. For example, survival of adenocarcinoma cells was reduced from 52 to 28 % in case of incubation with the UDD-DOX (Fig. 9, groups 1–4, UDD-DOX MCF-7) and from 72 to 30 % in case of incubation with OLC-DOX (Fig. 9, groups 1–4, OLC-DOX MCF-7). Hepatocellular carcinoma cells (HT29) appeared to be less sensitive to DOX. Increasing concentrations of trypsin and NCPCs-DOX correlated with decreasing of cell viability from 69 to 56 % in case of incubation with the UDD-DOX (Fig. 9, groups 1–4, UDD-DOX, HT29) and from 91 to 75 % in case of incubation with OLC-DOX (Fig. 9, groups 1–4, OLC-DOX, HT29).

**Table 3:** Composition of culture medium in experimental groups

	DMEM, μl	PBS, μl	
Control 0	100	0	
Control group 1	50	62.5	
Control group 2	50	75.0	
Control group 3	50	130	
Control group 4	50	220	
	DMEM, μl	CNMs-DOX, μg/ml	Trypsin, μg/ml
Trypsin control group 1	100	0	31.25
Trypsin control group 2	100	0	62.5
Trypsin control group 3	100	0	125
Trypsin control group 4	100	0	250
Group 1	50	84–2.5	31.25
Group 2	50	167–5	62.5
Group 3	50	335–10	125
Group 4	50	670–20	250

One of the main goals of this experiment was to test methods of synthesis of antitumor nanocarbon-protein conjugates with specific antibodies to the tumor receptors epidermal growth factors and metabolic drug. Another aim was to approbate methodology of controlled releasing of DOX in tumor cell culture. Unique physical and chemical properties of carbon nanomaterials are complemented by extremely interesting biological properties. Onion-like carbons (OLCs) and ultra dispersed diamonds (UDDs) have great potential for application in biology, medicine, and biotechnology [37–39]. There were many efforts to employ carbon nanomaterials as tool for anticancer therapy [25, 40–45]. But there are a lot of unclear aspects of CNM interaction with tumor cells. Toxicity and carcinogenicity has been one of the major concerns for OLCs and UDDs use in biomedical application [46, 47]. Our previous studies showed low cytotoxicity and good biocompatibility of UDDs and OLCs [26]. At the same time, we have found that the UDDs and OLCs can act as centers for cell cohesion and migrating in suspension. In this case CNMs can stimulate metastasis formation. Nevertheless, we suggested that UDDs and OLCs may be successfully functionalized by drug, such as DOX. Practically effective ammonization of carbon surface and high-sorption activity provided rather good functionalization of UDDox and OLCox by DOX. In addition, data from Hua et al. [48] and Kopecek et al. [49] demonstrated that drugs could be successfully released from carbon compounds. And, effectiveness of this process depends on the type of established connection. It was suggested that proteolysis enzymes such as trypsin will destroy covalent protein bonds in NCPCs. The concentration-dependent DOX release from UDD/OLC-DOX particle was realized by trypsin peptide bond decomposition. Correlation between concentration of trypsin and the amount of free DOX was typical for enzymatic reaction with substrate [50, 51]. It is indicated that release of DOX from NCPCs with UDDs and OLCs have enzymatic dose-dependent mechanism. This fact let us concluded that such proteolysis enzymes as pepsin, chemotropism, enterokinase, and elastase may be perspective for further decomposition of covalent peptide bonds in nanovehicles. The presence of these enzymes in gastric and intestinal secrets in normal and over secretion in cancer tissue [34] could provide natural cleavage of drugs from the carbon substrate in the stomach and intestines [36]. This process will provide targeted penetration of the drug into the zone of formation of colon or stomach cancer. Problem of targeted release of drugs can be resolved in another way. For examples, there are some studies of coating of nanodiamonds with PEG, silica, and polymetacrylate copolymers. The polymers enable to saturate the matrix by biologically active growth factors and cytokines with followed enzyme-dependent release in tissues [52]. Another functionalization of polymeric materials was demonstrated by Ehrbar with colleagues [53]. This work focused on tissue repair and not to its destruction, as is the case with the tumor. Nevertheless, the modification of nanostructured materials by their encapsulation in polymeric materials and further saturation with antitumor drugs and specific antibodies seems to us promising area of research [54]. Such constructions allow to modulate the drug release conditions according to the target tissue and the activity of its enzyme and tumor marker. Another significant problem of targeted delivery of drugs is the placement on the surface of the carbon particle-specific antibodies. Tumor cells have a specific receptor profile. These properties are the base for the diagnosis, prognosis of the disease, and development of treatment regimens for cancer patients. Thus, the “anti-receptor” drugs rather expensive and require the use of high doses. DOX as well as other antimetabolites and cytotoxic drugs have a wide range of harmful side effects on the body. Both referred approaches have advantages and disadvantages that limited the therapeutic application of drugs. For effective combination of cytotoxic and receptor-attractive approach, we use MAPLE technology of protein deposition. Early was reported that laser evaporation technique let safe activity of protein on rather high level [55, 56]. There are a lot of works with goals to attach specific antibodies to matrix [57, 58]. The main challenge in this work was to safe biological activity of antibody to EGF. Our results for the antibody activity are quite optimistic. Activity of anti-EGF antibodies was on the level 75 % after MAPLE deposition and in PBS. For testing antibody activities, ELISA method was used. Our results may be also verified by immune histochemistry methods with samples C37 and C47 and fluorescence microscopy. In fact, the inclusion of IHC and microscopy with high resolution would enable to confirm once more effectiveness of MAPLE technology for protein. In addition, fluorescence microscopy allows to observe distribution of NCPCs and its particles in cells. Another limitation of our work is that our research focuses only on cytological influence and characteristics of NCPCs, whereas it might be important to include more chemical characteristics of NCPCs. It is in our future plans.

## Conclusions

Synthesis of carbon protein construct on the basis of ultra dispersed diamond, onion-like carbon, anticancer drug (DOX), and anti-EGFR antibodies demonstrated fruitful cooperation of chemistry and biology. A new DOX delivery vehicle has been developed by step-by-step synthesis, cleaning, oxidation, functionalization with oxygen containing acidic groups (carboxylic—COOH, hydroxyl—OH), and immobilization of DOX on carbon surface of UDDox and OLCox. The effectiveness of functionalization was 2.94 % *w*/*w* for OLC-DOX and 2.98 % *w*/*w* for UDD-DOX. Here, dose-dependent, stepped-like increasing concentration of free DOX after incubation UDD-DOX and OLC-DOX conjugates with trypsin was described. It is demonstrated the effectiveness of trypsin as DOX release agent and UDD/OLC-DOX particles as drug delivery nanosystem. In addition in this paper, it has been shown that trypsin-mediated release of DOX has dose-dependent negative correlation with tumor cell viability. The survival of adenocarcinoma cells (MCF-7) reduced from 52 to 28 % in the case of incubation during 24 h with the UDD-DOX in concentrations from 8.4–2.5 μg/ml to 670–20 μg/ml and from 72 to 30 % after incubation with OLC-DOX at the same time and same concentrations. Sensitivity of hepatocellular carcinoma cells (HT29) to DOX was higher than MCF-7. This data illustrated that looking for appropriate drugs and system of delivery may be very complicated process even for cells of same epithelial genesis. For this purpose, possibility of deposition of specific anti-EGF antibodies of carbon surface by MAPLE technology was tested. Biological activity of anti-EGF antibodies after MAPLE deposition and PBS was near 75 %, which depended on technology of deposition and type of carbon film. Thus, on the basis of these results, we assume that NCPCs on the bases of UDDs and OLCs loaded with drugs and specific antibodies may be useful for effective antitumor agent delivery.

## References

[1] Schrand AM, Hens Ciftan SAC, Shenderova OA (2009). Nanodiamond particles: properties and perspectives for bioapplications. Crit Rev Solid State Mater Sci.

[2] Neugart F, Zappe A, Jelezko F (2007). Dynamics of diamond nanoparticles in solution and cells. Nano Lett.

[3] Sethuraman A, Stroscio MA, Dutta M, Stroscio MA, Dutta M (2004). Potential application of carbon nanotubes in bioengineering. Biological Nanostructures and Applications of Nanostructures in Biology. Electrical, Mechanical, and Optical Properties.

[4] Hasirci N, Mozafari MR (2007). Micro and nano systems in biomedicine and drug delivery. Nanomaterials and nanosystems for biomedical Applications.

[5] Katar S, Labiosa A, Plaud AE, Mosquera-Vargas E, Fonseca L, Weiner BR, Morell G (2009). Silicon encapsulated carbon nanotubes. Nanoscale Res Lett.

[6] Segura RA, Contreras C, Henriquez R, Häberle P, Acuña JJS, Adrian A, Alvarez P, Hevia SA (2014). Gold nanoparticles grown inside carbon nanotubes: synthesis and electrical transport measurements. Nanoscale Res Lett.

[7] Kurantowicz N, Strojny B, Sawosz E, Jaworski S, Kutwin M, Grodzik M, Wierzbicki M, Lipińska L, Mitura K, Chwalibog A (2015). Biodistribution of a high dose of diamond, graphite, and graphene oxide nanoparticles after multiple intraperitoneal injections in rats. Nanoscale Res Lett.

[8] Yellepeddi VK, Kumar A, Palakurty S (2009). Biotinylated poly(amido)amine (PAMAM) dendrimers as carriers for drug delivery to ovarian cancer cells in vitro. Anticancer Res.

[9] Chi-Cheng F, Lee H-Y, Chen K (2007). Characterization and application of single fluorescent nanodiamonds as cellular biomarkers. Proc Natl Acad Sci U S A.

[10] Schrand AM, Lin JB, Shenderova OA, Gruen DM (2006). Characterization of detonation nanodiamonds for biocampatibilty. Characterisation of Detonation Nanodiamonds.

[11] Liang X-J, Chen C, Zhao Y (2008). Biopharmaceutics and therapeutic potential of engineered nanomaterials. Curr Drug Metab.

[12] Tseng C-L, Chen K-H, Su W-Y (2013). Cationic gelatin nanoparticles for drug delivery to the ocular surface: in vitro and in vivo evaluation. J Nanomaterials.

[13] de Salamanca AE, Diebold Y, Calonge M (2006). Chitosan nanoparticles as a potential drug delivery system for the ocular surface: toxicity, uptake mechanism and in vivo tolerance. Invest Ophthalmol Vis Sci.

[14] Kaul G, Amiji M (2005). Tumor-targeted gene delivery using poly(ethylene glycol)-modified gelatin nanoparticles: in vitro and in vivo studies. Pharm Res.

[15] Goldberg MS, Hook SS, Wang AZ (2005). Biotargeted nanomedicines for cancer: six tenets before you begin nanomedicine. Nanomedicine.

[16] Praveena M, Rapoport N (2010). Doxorubicin as a molecular nanotheranostic agent: effect of doxorubicin encapsulation in micelles or nanoemulsions on the ultrasound-mediated intracellular delivery and nuclear trafficking. Mol Pharm.

[17] Kamba SA, Ismail M, Hussein-Al-Ali SH (2013). In vitro delivery and controlled release of doxorubicin for targeting osteosarcoma. Molecules.

[18] Ottewell PD, Jones M, Lefley DV (2008). Antitumor effects of doxorubicin followed by zoledronic acid in a mouse model of breast cancer. J Natl Cancer Inst.

[19] Medina SH, Chevliakov MV, Tiruchinapally G (2013). Enzyme-activated nanoconjugates for tunable release of doxorubicin in hepatic cancer cells. Biomaterials.

[20] Thorna CF, Oshiroa C, Marshe S, Hernandez-Boussardb T, Hd ML, Kleina TE, Altmana RB (2011). Doxorubicin pathways: pharmacodynamics and adverse effects. Pharmacogenet Genomics.

[21] Payne S, Miles D, Gleeson M, Browning G, Burton J, Clarke R, Hibbert J, Jones NS (2008). Mechanisms of anticancer drugs. Scott-Brown’s Otorhinolaryngology: Head and Neck Surgery 7Ed.

[22] Tseng C-L, Su W-Y, Yen K-C (2009). The use of biotinylated-EGF-modified gelatin nanoparticle carrier to enhance cisplatin accumulation in cancerous lungs via inhalation. Biomaterials.

[23] Nagarwal RC, Kant S, Singh PN, Maiti P, Pandit JK (2009). Polymeric nanoparticulate system: a potential approach for ocular drug delivery. J Control Release.

[24] Mou’ad AT and Sahrim HA. Characterization and morphology of modified multi-walled carbon nanotubes filled thermoplastic natural rubber (TPNR) composite syntheses and applications of carbon nanotubes and their composites. Nanotechnology and Nanomaterials. 2013. doi:10.5772/50726.

[25] Ashwin AB, Vyomesh P, Julie G (2009). Targeted killing of cancer cells *in vivo* and *in vitro* with EGF-directed carbon nanotube-based drug delivery. ACS Nano.

[26] Yakymchuk OM, Perepelytsina OM, Rud AD (2014). Impact of carbon nanomaterials on the formation of multicellular spheroids by tumor cells. Phys Status Solidi A.

[27] Dai X, Eccleston M, Swartling J, Slater N (2008). Fluorescence and lifetime imaging of free and micellarencapsulated doxorubicin in living cells. Nanomedicine.

[28] Piqué A, McGill RA, Chrisey DB (1999). Growth of organic thin films by the matrix assisted pulsed laser evaporation (MAPLE) technique. Thin Solid Films.

[29] Hunter CN, Check MH, Bultman JE (2008). Development of matrix-assisted pulsed laser evaporation (MAPLE) for deposition of disperse films of carbon nanoparticles and gold/nanoparticle composite films. Surf Coat Technol.

[30] Califano V, Bloisi F, Vicari LRM (2009). Matrix assisted pulsed laser evaporation (MAPLE) of poly(D, L lactide) (PDLLA) on three dimensional bioglass structures. Adv Eng Mat.

[31] Caricato AP, Luches A, Rella R (2009). Nanoparticle thin films for gas sensors prepared by matrix assisted pulsed laser evaporation. Sensors.

[32] Smausz T, Megyeri G, Kékesi R (2009). Functionalized polysiloxane thin films deposited by matrix-assisted pulsed laser evaporation for advanced chemical sensor applications. Thin Solid Films.

[33] Mosmann T (1983). Rapid colorimetric assay for cellular growth and survival: application to proliferation and cytotoxic assay. J Immunol Methods.

[34] Syed R, Farukh R, Shaista R (2012). Role of proteases in cancer: a review. Biotechnol Mol Biol Rev.

[35] Skitzki JJ, Chang AE (2002). Hepatic artery chemotherapy for colorectal liver metastases: technical considerations and review of clinical trials. Surg Oncol.

[36] Yamada R, Kishi K, Sato M (1995). Transcatheter arterial chemoembolization (TACE) in the treatment of unrectable liver cancer. World J Surg.

[37] Bartelmess J, Giordani S (2014). Carbon nano-onions (multi-layer fullerenes): chemistry and applications. Beilstein J Nanotechnol.

[38] Fu Fu CC (2007). Characterization and application of single fluorescent nanodiamonds as cellular biomarkers. Proc Natl Acad Sci U S A.

[39] Mochalin VN, Shenderova O, Gogotsi Y (2012). The properties and applications of nanodiamonds. Nat Nanotechnol.

[40] Li L, Tang F, Liu H (2010). In vivo delivery of silica nanorattle encapsulated docetaxel for liver cancer therapy with low toxicity and high efficacy. ACS Nano.

[41] Liu S-Y, Liang Z-S, Gao F (2010). In vitro photothermal study of gold nanoshells functionalized with small targeting peptides to liver cancer cells. J Mater Sci Mater Med.

[42] Maeng JH, Lee DH, Jung KH (2010). Multifunctional doxorubicin loaded superparamagnetic iron oxide nanoparticles for chemotherapy and magnetic resonance imaging in liver cancer. Biomaterials.

[43] Na K, Park K-H, Kim SW, Bae YH (2000). Self-assembled hydrogel nanoparticles from curdlan derivatives: characterization, anti-cancer drug release and interaction with a hepatoma cell line (HepG2). J Control Release.

[44] Maeda H (2001). SMANCS and polymer-conjugated macromolecular drugs: advantages in cancer chemotherapy. Adv Drug Deliv Rev.

[45] Duncan R, Seymour LW, O’Hare KB (1992). Preclinical evaluation of polymer-bound doxorubicin. J Control Release.

[46] Baidakova M, Vul A (2007). New prospects and frontiers of nanodiamond clusters. J of Physics D Appl Phys.

[47] Chiganov AS (2004). Selective inhibition of the oxidation of nanodiamonds for their cleaning. Phys Solid State.

[48] Hua H, Lien Ai P-H, Pierre D (2013). Carbon nanotubes: applications in pharmacy and medicine. BioMed Res Int.

[49] Kopecek J, Kopecková P, Minko T, Lu ZR (2000). HPMA copolymereanticancer drug conjugates: design, activity, and mechanism of action. Eur J Pharm Biopharm.

[50] David A, Kopecková P, Rubinstein A, Kopecek J (2001). Enhanced biorecognition and internalization of HPMA copolymers containing multiple or multivalent carbohydrate side-chains by human hepatocarcinoma cells. Bioconjug Chem.

[51] Tang A, Kopecková P, Kopecek J (2003). Binding and cytotoxicity of HPMA copolymer conjugates to lymphocytes mediated by receptor-binding epitopes. Pharm Res.

[52] Rehor I, Mackova H,Sergey K Filippov, Kucka J, Proks V, Slegerova J, Turner S, Gustaaf Van Tendeloo, Ledvina M, Hruby M, Cigler P. Fluorescent nanodiamonds with bioorthogonally reactive protein-resistant polymeric coatings. ChemPlusChem. 2013; doi: 10.1002/cplu.201300339. 10.1002/cplu.201300339 31986754

[53] Ehrbar M, Djonov VG, Schnell C, Tschanz SA, Martiny-Baron G, Schenk U, Wood J, Burri PH, Hubbell JA, Zisch Lkjh AH (2004). Cell-demanded liberation of VEGF121 from fibrin implants induces local and controlled blood vessel growth. Circ Res.

[54] de la Cruz EF, Zheng Y, Torres E, Li W, Song W, Burugapalli K (2012). Zeta potential of modified multi-walled carbon nanotubes in presence of poly (vinyl alcohol) hydrogel. Int J Electrochem Sci.

[55] Bloisi F, Vicari RML, Papa R (2007). Biomaterial thin film deposition and characterization by means of MAPLE technique materials science and engineering. Materials Sci Eng C.

[56] Bloisi F, Pezzella A, Vicari RML, Barra M. Matrix assisted pulsed laser deposition of melanin thin films. J of applied Physics. 2011; doi:10.1063/1.3602084.

[57] Daad A Abi-Ghanem and Berghman Luc R. Immunoaffinity chromatography: a review. http://cdn.intechopen.com/pdfs-wm/33050.pdf. Accessed 15 Jun 2015.

[58] Alberts B, Jonson A, Lewis J, Alberts B, Johnson A, Lewis J, Raff M, Roberts K, Walter P (2002). Manipulating proteins, DNA and RNA. Molecular biology of the cell.

